# Ureterocolic fistula as an incidental finding after barium enema

**DOI:** 10.1590/0100-3984.2017.0193

**Published:** 2019

**Authors:** Michael Frank Pereira

**Affiliations:** 1 Universidade Federal do Espírito Santo (UFES), Vitória, ES, Brazil

**Figure 1:**
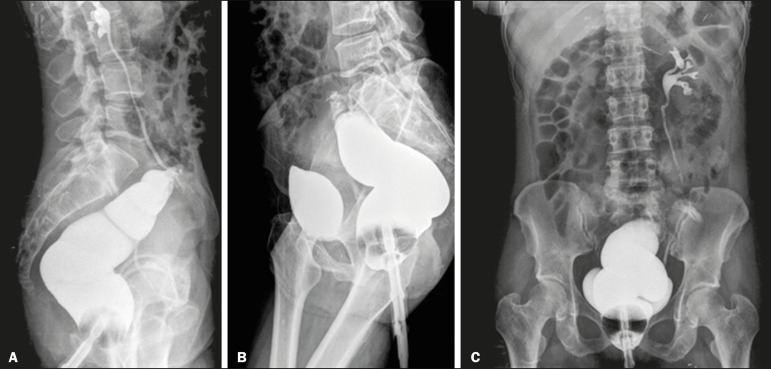
X-rays obtained after barium enema. **A:** Lateral view showing initial opacification of the rectum, remaining distal colon, ureter, and left renal collecting system. **B:** Lateral view showing late opacification of the bladder. **C:** Posteroanterior view showing late opacification of the bladder.

Dear Editor,

A 42-year-old female patient who had undergone Hartmann’s procedure for the treatment of colorectal carcinoma 7 months prior presented for preoperative evaluation before closure of the colostomy. She reported no clinical symptoms or comorbidities and stated that she had never received chemoradiotherapy. She was given a barium enema (Figure 1), after which there was opacification of the ureter and left renal collecting system, consistent with ureterocolic fistula. Although the fistulous tract was difficult to characterize, it appeared to be connecting the distal stump to the middle third of the left ureter. There was also late opacification of the bladder. Careful image analysis is important, in order to avoid an incorrect diagnosis of enterovesical fistula with vesicoureteral reflux.

Ureterocolic fistulas are rare and have a variety of causes, most being urological^(1)^ or iatrogenic in origin, with an inflammatory, neoplastic, or idiopathic etiology. The recent increase in the number of ureteroscopic and laparoscopic procedures has greatly increased the incidence of ureterocolic fistulas^(2)^, because surgical manipulation generates inflammation that affects the ureter and leads to their formation^(3)^. Anatomically, most cases involve the right ureter, in its upper and middle thirds, rarely occurring on the left^(1,4,5)^.

The most common symptom of ureterocolic fistula is nonspecific abdominal pain, typically on the flanks. Peritoneal irritation with psoas muscle involvement can lead to Morton’s triad, consisting of low back pain, thigh adduction, and lower limb flexion. As a rule, there are no digestive symptoms, although some patients present with pneumaturia or fecaluria^(6)^.

Ureterocolic fistula can be diagnosed on contrast-enhanced examinations, such as X-rays obtained after a barium enema and retrograde cystourethrography^(7)^. Obtaining X-ray images after administration of a barium enema is the most reliable method of identifying the fistulous tract^(8)^. Although retrograde voiding cystourethrography can allow visualization of the fistulous tract, it can be difficult to identify the ureteral meatus with the method, because of the surrounding edema attributed to the inflammatory process and because of obstruction of the ureteral pathway by the fistula. Computed tomography is the most sensitive method for identifying pneumaturia and the fistulous tract.

The treatment of ureterocolic fistula consists in the surgical removal of the fistula. The technique employed varies depending on which portion of the ureter is affected, as well as on whether there is any accompanying renal dysfunction.

In the case reported here, the emergence of the ureterocolic fistula was iatrogenic, being attributed to previous surgical manipulation. A contrast-enhanced imaging examination was essential because it allowed the fistula to be corrected during the surgical procedure that was being planned, as well as allowing the patient to be referred to the nephrology department for the clinical monitoring of any renal dysfunction that might develop.
